# Data Analysis Strategies for Microbiome Studies in Human Populations—a Systematic Review of Current Practice

**DOI:** 10.1128/mSystems.01154-20

**Published:** 2021-02-23

**Authors:** Sven Kleine Bardenhorst, Tom Berger, Frank Klawonn, Marius Vital, André Karch, Nicole Rübsamen

**Affiliations:** a Institute of Epidemiology and Social Medicine, University of Münster, Münster, Germany; b Research Group Biostatistics, Helmholtz Centre for Infection Research, Braunschweig, Germany; c Institute of Medical Microbiology and Hospital Hygiene, Hannover Medical School, Hannover, Germany; KU Leuven

**Keywords:** microbiome, 16S rRNA, shotgun metagenomics sequencing, analysis strategies

## Abstract

Reproducibility is a major issue in microbiome studies, which is partly caused by missing consensus about data analysis strategies. The complex nature of microbiome data, which are high-dimensional, zero-inflated, and compositional, makes them challenging to analyze, as they often violate assumptions of classic statistical methods. With advances in human microbiome research, research questions and study designs increase in complexity so that more sophisticated data analysis concepts are applied. To improve current practice of the analysis of microbiome studies, it is important to understand what kind of research questions are asked and which tools are used to answer these questions. We conducted a systematic literature review considering all publications focusing on the analysis of human microbiome data from June 2018 to June 2019. Of 1,444 studies screened, 419 fulfilled the inclusion criteria. Information about research questions, study designs, and analysis strategies were extracted. The results confirmed the expected shift to more advanced research questions, as one-third of the studies analyzed clustered data. Although heterogeneity in the methods used was found at any stage of the analysis process, it was largest for differential abundance testing. Especially if the underlying data structure was clustered, we identified a lack of use of methods that appropriately addressed the underlying data structure while taking into account additional dependencies in the data. Our results confirm considerable heterogeneity in analysis strategies among microbiome studies; increasingly complex research questions require better guidance for analysis strategies.

**IMPORTANCE** The human microbiome has emerged as an important factor in the development of health and disease. Growing interest in this topic has led to an increasing number of studies investigating the human microbiome using high-throughput sequencing methods. However, the development of suitable analytical methods for analyzing microbiome data has not kept pace with the rapid progression in the field. It is crucial to understand current practice to identify the scope for development. Our results highlight the need for an extensive evaluation of the strengths and shortcomings of existing methods in order to guide the choice of proper analysis strategies. We have identified where new methods could be designed to address more advanced research questions while taking into account the complex structure of the data.

## INTRODUCTION

Recent advances in high-throughput sequencing methods led to an exponentially increasing number of publications that aim to investigate the relationship between diseases and structural changes in the human microbiome (1–3). Reproducibility remains a major issue in this context. While some publications find support for a link between the microbiome and a disease, other studies often lead to different or even contradictory conclusions. This conflict can be exemplified by a recent review on the role of the gut microbiome in Parkinson’s disease which shows that among 16 studies comparing gut microbiota between Parkinson’s disease patients and healthy individuals, 100 different taxa were detected to be differentially abundant (4). Notably, several taxa, e.g., *Lactobacillaceae* and *Bacteroidetes*, were significantly increased in Parkinson’s disease patients in four studies but significantly decreased in two other studies.

Heterogeneity between studies and low reproducibility may be caused by many sources of variability in microbiome data. While biological differences exist, e.g., based on the genetics of the host or its diet, there is a lot of potential for technical variation in microbiome studies. Technical variation may be introduced during sequencing as well as by the bioinformatics pipeline used to translate the results of the sequencing into the composition of the microbiome (5). The choice of the data analysis strategies following this process also contributes to the observed heterogeneity. Issues in microbiome data prohibit the use of classic statistical methods, especially methods designed for low-dimensional data that make specific assumptions about the data, which do not hold in the microbiome context. Microbiome data obtained by 16S rRNA amplicon or shotgun metagenomic sequencing are high dimensional, with thousands of taxa present. In addition, microbiome data are sparse because specific taxa are either not present in some samples (structural zeros) or are not detected due to low abundance (technical zeros). This is especially problematic because microbiome data are compositional and add up to a fixed overall read number (6–8), which in itself is variable and mainly determined by technical issues and not the true quantity of microbiota in the original sample. Taxa with low abundance are more likely to be considered absent in samples with a low number of total reads, which will lead to bias if analysis strategies are based on relative frequencies (9).

As research in the field advances, study designs become more complex and need appropriate analysis strategies. While many early publications focused on the characterization of different parts of the human microbiome in healthy individuals or in the context of diseases, recent publications focus on more distinguished links between the microbiome and diseases, e.g., the detection of predictive biomarkers that may enable early diagnosis of diseases or the effect of a disease on the development of the microbiome over time.

This review aims to identify recent studies with a focus on microbiota in the human host and to extract information about what kind of research questions are asked, which study designs are used to answer these questions, and which statistical methods are applied to analyze the data. The results will provide an overview of current practice in microbiome studies and highlight the challenges posed by the complex data structure of microbiome data.

## RESULTS

### Research questions and study designs.

Out of the 419 studies evaluated in this review (see Fig. S2 in the supplemental material), 307 (73%) collected microbiome samples of individuals from a single time point using a cross-sectional design. The majority (98.1%) of these studies assessed the outcome at the same time as the exposure; six studies (1.9%) used a time-to-event analysis (with microbiome data as the exposure of interest). Among studies that sampled at a single time point, 14% obtained clustered data, e.g., by sampling multiple body sites of the same individual. A total of 112 studies (27%) collected repeated microbiome samples of the same individual using a longitudinal study design.

Inconsistencies between the objectives and what was achieved through analysis could be identified (Table 1) in 31.7% of the analyzed studies. Most studies claimed analytical objectives (*n* = 316, 75.4%); however, some of these studies (19.6%) either built predictive models to assess predictive performance or tested for explicitly defined treatment effects. In contrast, 39.3% of the 28 studies claiming predictive objectives did not actually do this but instead performed only group comparisons—either descriptive or based on statistical tests—without any measure of predictive performance. Most studies that claimed descriptive goals actually performed statistical tests to detect group differences (90.9% out of *n* = 44). Among the 31 studies that aimed to investigate treatment effects, 17 (54.8%) did not formally assess treatment effects but only performed statistical tests to compare the microbial community structure between study groups.

**TABLE 1:** Comparison of the intended objective with the performed analysis^*a*^

Objective	Actual analysis			Treatment effect	Row total
	Descriptive	Analytical	Predictive		
Descriptive	4	39	1	0	44
Analytical	12	260	43	1	316
Predictive	1	10	17	0	28
Treatment effect	0	14	3	14	31
Column total	17	323	64	15	419

a Cell counts represent number of studies.

### Sample size.

**FIG 1:**
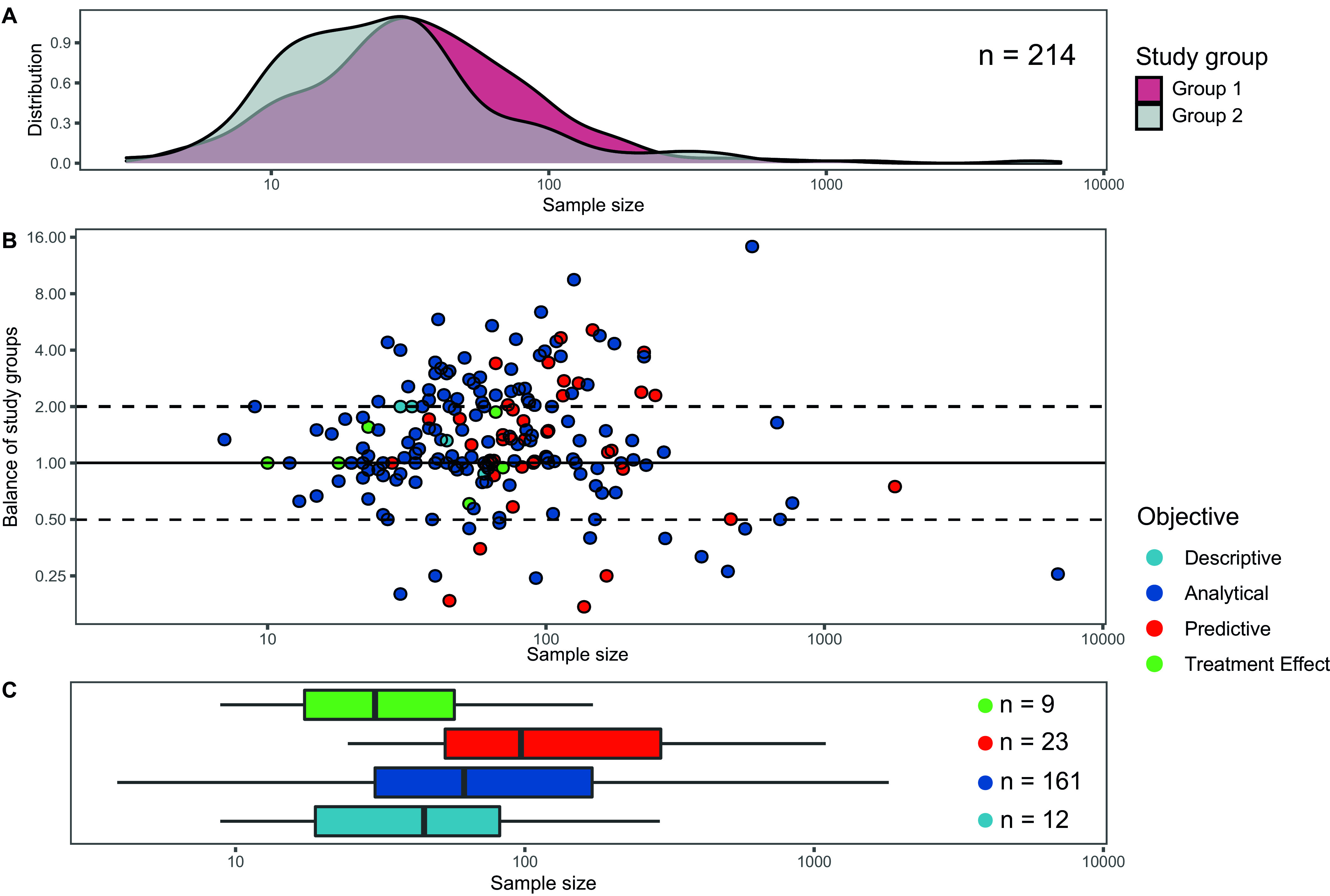
Sample sizes and balances of study groups. (A) Distribution of sample size stratified by study group. *n* refers to the number of studies with two study groups. (B) Balance of study groups stratified by research objective. The vertical axis indicates the balance in sample size between study groups, with one representing equal group sizes. Points outside the dashed lines indicate studies in which one study group is at least twice as large as the second study group. Note, only studies with two study groups are presented here. (C) Distribution of sample sizes for studies using only one study group.

Among all studies included, 51.1% (*n* = 214) compared two study groups, typically a group of individuals with the outcome of interest (further referred to as group 1) and a group without (further referred to as group 2). Two-hundred and five (48.9%) studies sampled one study group, usually within longitudinal study designs or for subgroup comparisons within the study group of interest. Studies using only 1 study group had a median sample size of 51 ranging from 3 to 1,709. Studies with 2 study groups had a median sample size of 60.5 ranging from 7 to 6,896; the median size of study groups 1 and 2 were 32 and 24, respectively. Among all studies with 2 study groups, an unbalanced design was common, with group 1 being twice as big as group 2 (or vice versa) in 25.9% of the studies (Fig. 1B). The largest overall sample size could be observed in studies with analytical objectives. An in-depth overview of the samples sizes is depicted in Table 2.

**TABLE 2:** Sample sizes stratified by research objective and study groups for studies with two study groups

Objective	Study group	Min	Q1^*a*^	Median	Q3^*a*^	Max
Descriptive (*n* = 5)	Group 1	10	20	22	25	28
	Group 2	10	10	11	19	32
	Overall	20	30	33	44	60
Analytical (*n* = 162)	Group 1	4	18.25	31	59	1404
	Group 2	3	13	20	37.75	5492
	Overall	7	34	55	98.25	6896
Predictive (*n* = 41)	Group 1	7	32	48	88	767
	Group 2	14	29	35	48	1025
	Overall	28	65	84	137	1792
Treatment effect (*n* = 6)	Group 1	5	10.25	17	30.5	43
	Group 2	5	9	16	30.5	36
	Overall	10	19.25	38	62.75	70

a Q1, first quartile; Q3, third quartile.

### Software.

The statistical programming language R (48.6%) was used most frequently for the analysis of microbiome data and 2.2% used Python. Although mothur is a bioinformatic pipeline by nature, it incorporates functions to be used for statistical analysis of the processed data; they were applied in 14.8% of the studies. PICRUSt and cytoscape, both open source software packages, were used in 18.9% and 4.3% of the studies, respectively. SPSS was used by 16.2% of studies, while either Stata or SAS were used in 5.6% of the studies. Calypso, Galaxy, and metastats—analysis platforms designed for the analysis of microbiome data—were used by 7.2% of the studies. More than one-fourth of the studies (*n* = 117, 30.1%) did not explicitly mention the software used for analysis, suggesting that analyses were performed by the used bioinformatics pipeline. A detailed overview of the software used can be found in Fig. S2.

### Taxonomic levels.

**FIG 2:**
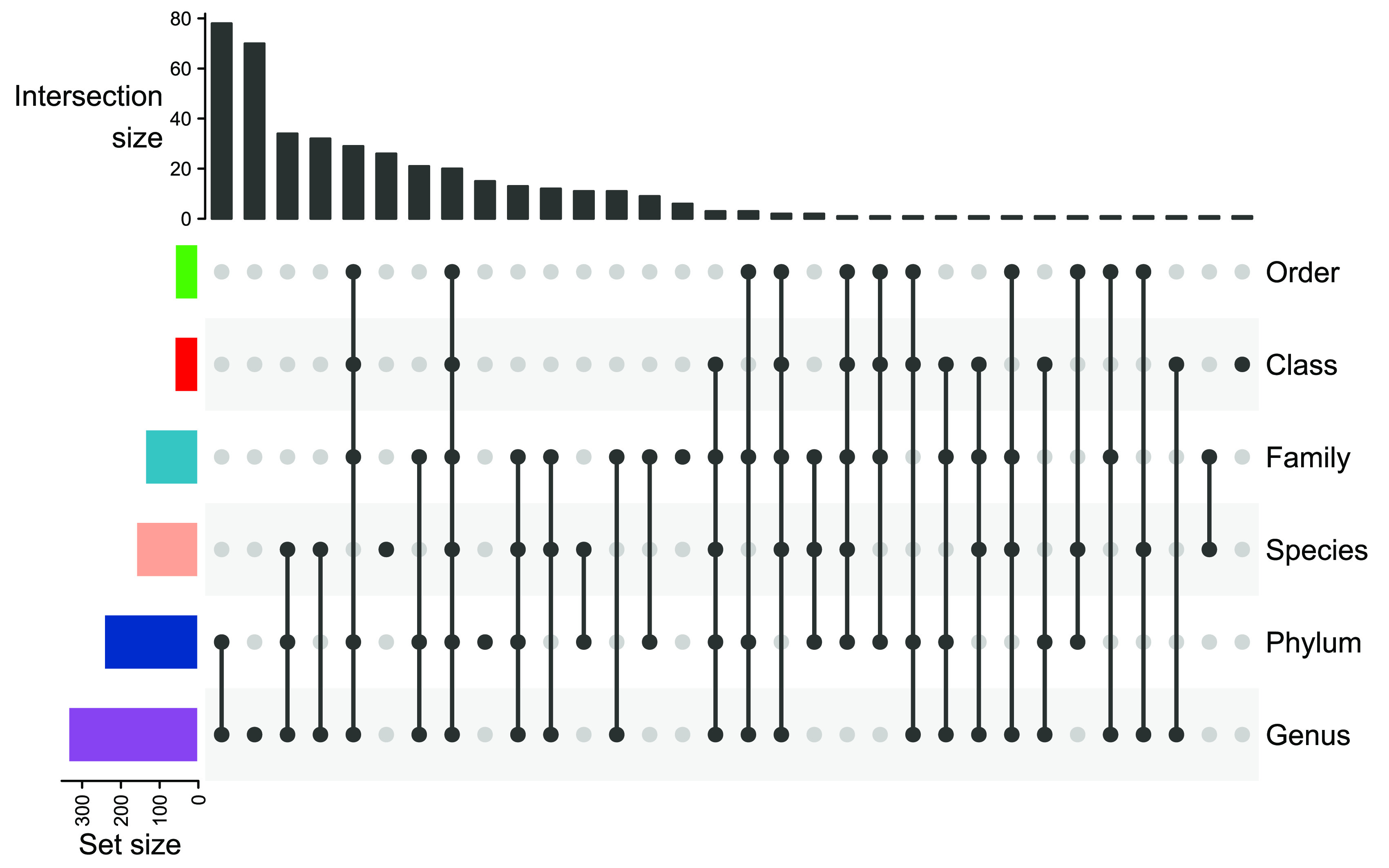
Upset plot of most frequently applied investigated combinations of taxonomic levels.

The majority of studies (96.9%) used the classical operational taxonomic unit (OTU) approach to cluster reads and assign taxonomic annotations to the clusters. Thirteen studies (3.1%) used the DADA2 (10) pipeline, indicating the use of amplicon sequence variants (ASVs), and assigned taxonomic annotation directly to sequence reads without previous clustering. Genus was the most frequently used level for analysis (75.7%), followed by phylum (55.3%). The species level was investigated by 34.7% of the studies; however, only 16.0% of the studies focused on species level data only. In general, 66.8% (*n *= 280) of all included studies performed analyses at multiple taxonomic levels, with a wide range of different combinations (Fig. 2). The most frequently investigated sets were genus and phylum (17.7%) and genus, phylum, and species (7.9%). Only 18 studies (4.3%) investigated all taxonomic levels.

### Alpha diversity analysis.

**FIG 3:**
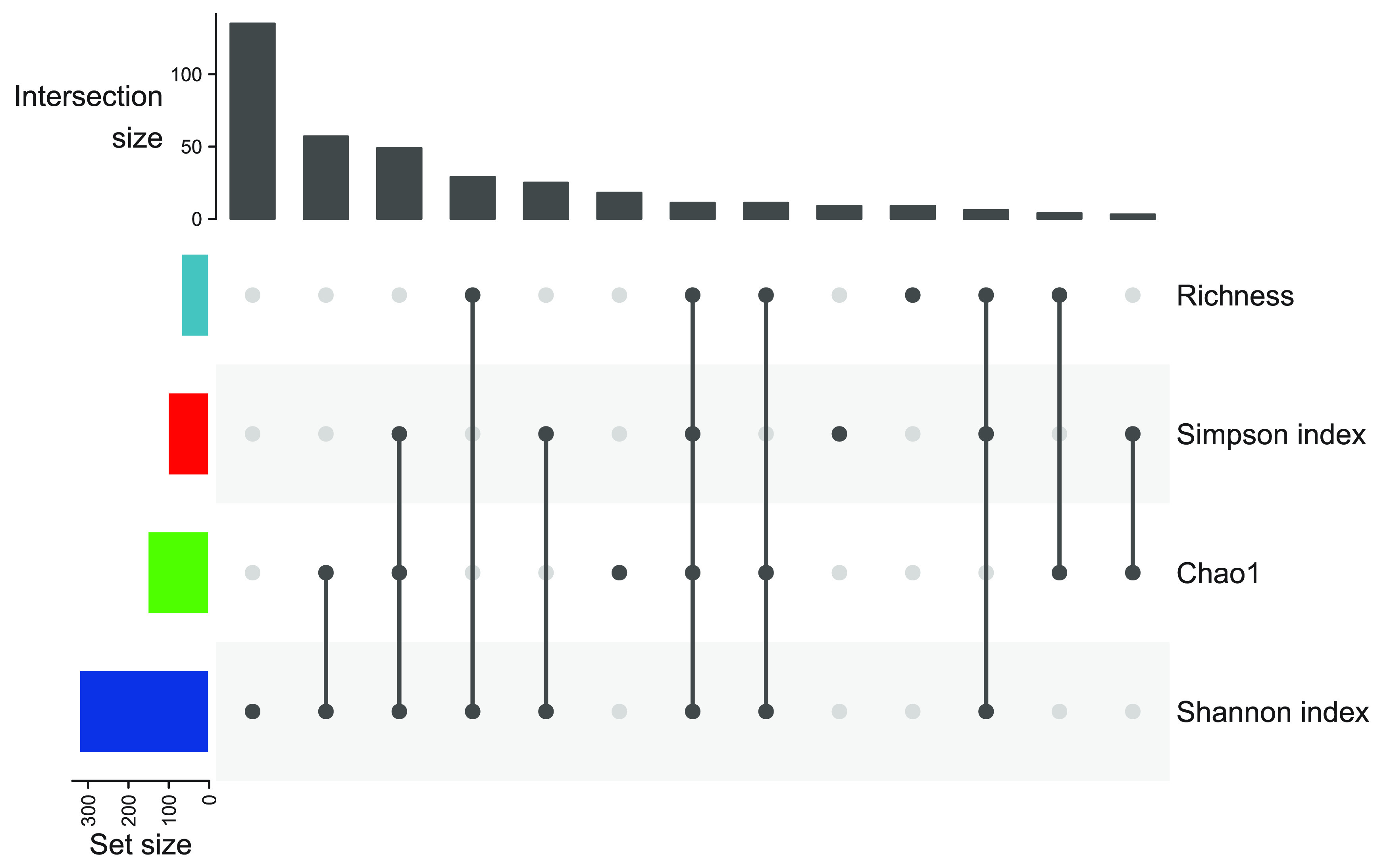
Upset plot of most frequently applied sets of alpha diversity measures.

Alpha diversity was investigated by 87.1% (*n *= 365) of all studies. Among these studies, 12 different indices were used to quantify alpha diversity, richness or eveness (11–15) (see Fig. S1 in the supplemental material). The Shannon index (16) was used most frequently (88.5%), followed by Chao1 (17) (41.9%), the Simpson index (18) (28.2%), and observed richness (19) (19.2%). A commonly observed strategy was to investigate a set of indices jointly (Fig. 3).

10.1128/mSystems.01154-20.1 FIG S1 Shows all observed alpha and beta diversity indices and how often these indices were used. Download FIG S1, EPS file, 1.8 MB. Copyright © 2021 Kleine Bardenhorst et al. 2021 Kleine Bardenhorst et al. https://creativecommons.org/licenses/by/4.0/ This content is distributed under the terms of the Creative Commons Attribution 4.0 International license.

10.1128/mSystems.01154-20.2 FIG S2 Shows the observed software and how often each software was used. Download FIG S2, EPS file, 1.1 MB. Copyright © 2021 Kleine Bardenhorst et al. 2021 Kleine Bardenhorst et al. https://creativecommons.org/licenses/by/4.0/ This content is distributed under the terms of the Creative Commons Attribution 4.0 International license.

**FIG 4:**
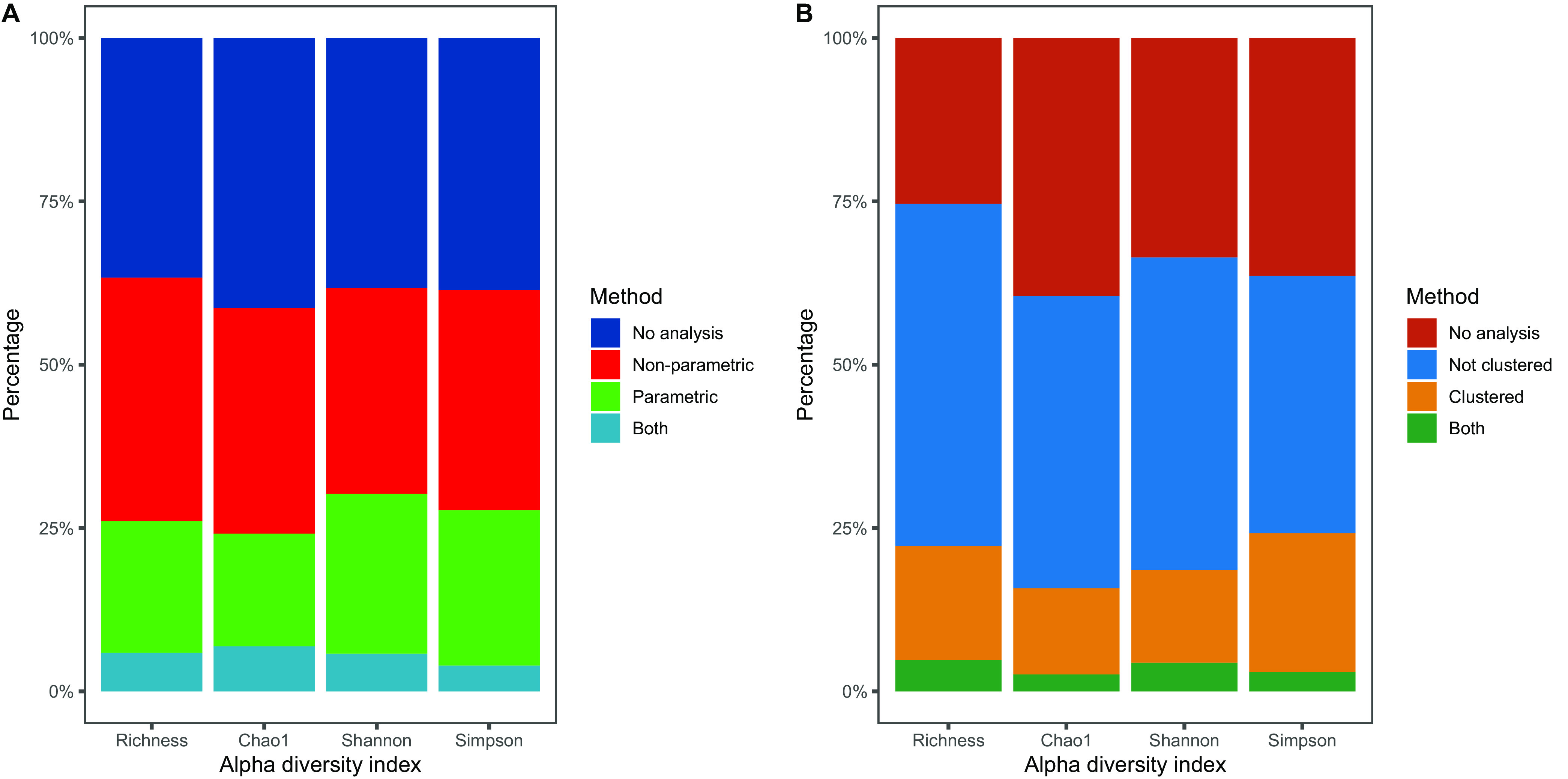
Analysis of most frequently used alpha diversity measures. (A) Proportion of studies testing for differences in the respective indices between groups by parametric methods, nonparametric methods. or both. (B) Proportion of studies that used methods designed for clustered data, not designed for clustered data, or both. A total of 100.0% refers to all studies that analyzed clustered data. Richness refers to observed species richness.

About 38% of the studies focused on an exploratory approach to alpha diversity only, comparing alpha diversity indices between groups without testing these differences statistically (Fig. 4). Among those studies that performed statistical tests (*n *= 227), 37% used parametric tests, while the rest used nonparametric tests or a combination of both parametric and nonparametric tests.

In the 134 (43.4%) studies with clustered data, only between 13.2% (Chao1) and 21.2% (Simpson index) of the alpha diversity analyses took clustering into account at the analysis stage (Fig. 4).

### Beta diversity analysis.

**FIG 5:**
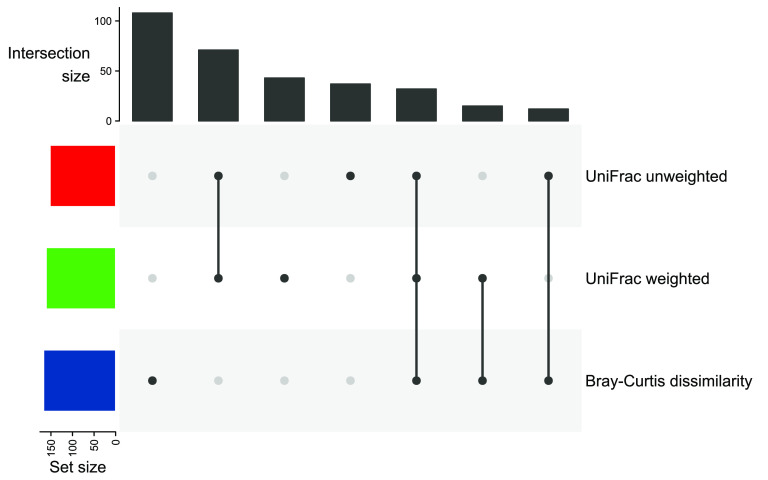
Upset plot of most frequently applied sets of beta diversity measures.

Beta diversity was investigated by 87.1% (*n *= 365) of all studies. The majority of studies used weighted (44.1% of studies investigating beta diversity) or unweighted (41.6%) UniFrac distance (20), followed by the Bray-Curtis dissimilarity (21) (45.8%). The remaining indices were used in less than 5.0% of the studies investigating beta diversity (22–26) (Fig. S1). Half of the studies that investigated beta diversity (51%, *n* = 186) focused on a single index, while 30.4% (*n *= 112) investigated two different indices and 9.6% (*n *= 35) more than two indices. UniFrac distances were predominantly investigated as a joint set, considering the weighted and unweighted version, or together with Bray-Curtis dissimilarity (Fig. 5). In the studies focusing on a single metric, Bray-Curtis dissimilarity was used dominantly.

The most frequently used approach to detect differences in beta diversity between groups was permutational multivariate analysis of variance (PERMANOVA) (27) (45.2%, *n* = 165), followed by analysis of similarity (ANOSIM) (28) (13.4%, *n* = 49). Violation of the assumption of heterogeneity of multivariate dispersion was generally not reported, even though unbalanced groups were quite common (Fig. 1). More than one-third (39%, *n* = 85) of the studies using PERMANOVA or ANOSIM analyzed clustered data, which can be accounted for by PERMANOVA or ANOSIM by restricted permutation schemes. However, as most studies did not report how these methods were implemented, it is not clear whether these adjustments were applied.

### Dimension reduction.

The most frequently used ordination method was principal-coordinate analysis (29) (PCoA) (63% of studies investigating beta diversity), while 10.8% (n = 37) used nonmetric multidimensional scaling (30) (NMDS). Classical principal-component analysis (PCA), applied either to the count or relative abundance data or to already transformed data, was used in 9.0% of the studies. Almost 7% of all studies assessing beta diversity used an unsupervised clustering approach to define groups with similar bacterial community structures. In these studies, the most frequently used method was Dirichlet multinomial mixtures (DMM) (31) (36%, *n* = 9), followed by partition around medoids (PAM; 28%, *n* = 7) (32) and k-means clustering (33) (12.0%, *n* = 3). Only DMM is applied to raw count data directly, while the other methods are based on the chosen beta diversity measure.

### Differential abundance analysis.

The largest amount of heterogeneity was found at the stage of differential abundance testing, with 45 different approaches used. About three-fourths of all studies in this review (77.1%) investigated differential abundance, while one-fourth (22.9%) focused solely on diversity analyses. As differential abundance testing is often performed univariately for every possible taxon, multiple testing need to be addressed. Among those studies that tested for differential abundance univariately, 58.1% corrected for multiple testing. Among those studies, 84.8% used the Benjamini-Hochberg false-discovery rate correction (34), while 15.2% used a Bonferroni correction (35). About half of the studies investigating differential abundance used a combination of multiple methods to test the same hypotheses (44.3%).

### Nonparametric differential abundance analysis.

Nonparametric methods were the most frequently applied group of methods for differential abundance testing (69.3% of all studies, *n* = 224) out of all the studies. Among these methods, linear discriminant analysis effect size (LEfSe) (36) (a sequence of nonparametric tests combined specifically for the microbiome research field) was used most commonly (58.9%), followed by the Mann-Whitney U test (37, 38) (22.2%) and the Kruskal-Wallis test (39) (20.8%). Analysis of composition of microbiomes (ANCOM), a method designed specifically for microbiome data under the framework of compositional data analyses (40), was applied in 13 studies (5.8%).

### Parametric differential abundance analysis.

Different types of parametric models were used for differential abundance analysis. Thirty-eight studies (11.8%) used simple parametric tests for group differences, e.g., ANOVA (47.4%) or *t* test (42.1%). Only one of these studies applied transformations (beyond relative abundance transformation) prior to analysis.

More often, generalized linear models (GLMs; 22.3%, *n* = 72) were used. Based on the type of GLM, the model either treats the microbiome data as independent or as a dependent variable.

### Parametric differential abundance analysis—microbiome as predictor.

Among studies (*n* = 155) treating the microbiome as independent variables, logistic (23.9%) and linear (32.4%) regression were used most frequently, followed by partial least-squares-discriminant analysis (PLS-DA; 19.4%) (41) and multivariate association with linear models (MaAsLin; 18.3%) (42). Among all studies that used generalized linear models with microbiome as the independent variable, 26.4% (*n* = 19) transformed their data prior to analysis; in particular, 13 studies used ArcSine square root transformations—transformations applied to relative abundance data (43) and part of the MaAsLin workflow—and five studies used centered log-ratio (44) (CLR) transformations. Five studies used Cox regression with microbiome data as independent variables.

### Parametric differential abundance analysis—microbiome as outcome.

Generalized linear models for count data model the microbiome as the dependent variable and were used by 5.6% (*n* = 18) of the studies. Among those studies, negative binomial (45) (77.8%) and Poisson (46) (21.6%) regression models were used most frequently, followed by their zero-inflated extensions (16.7% [47] and 11.1% [48], respectively). More elaborated negative binomial models as implemented by edgeR (49) or DESeq2 (50) were classified as GLMs as well, as they were reported as negative binomial models in many cases so that a clear distinction was not possible.

**FIG 6:**
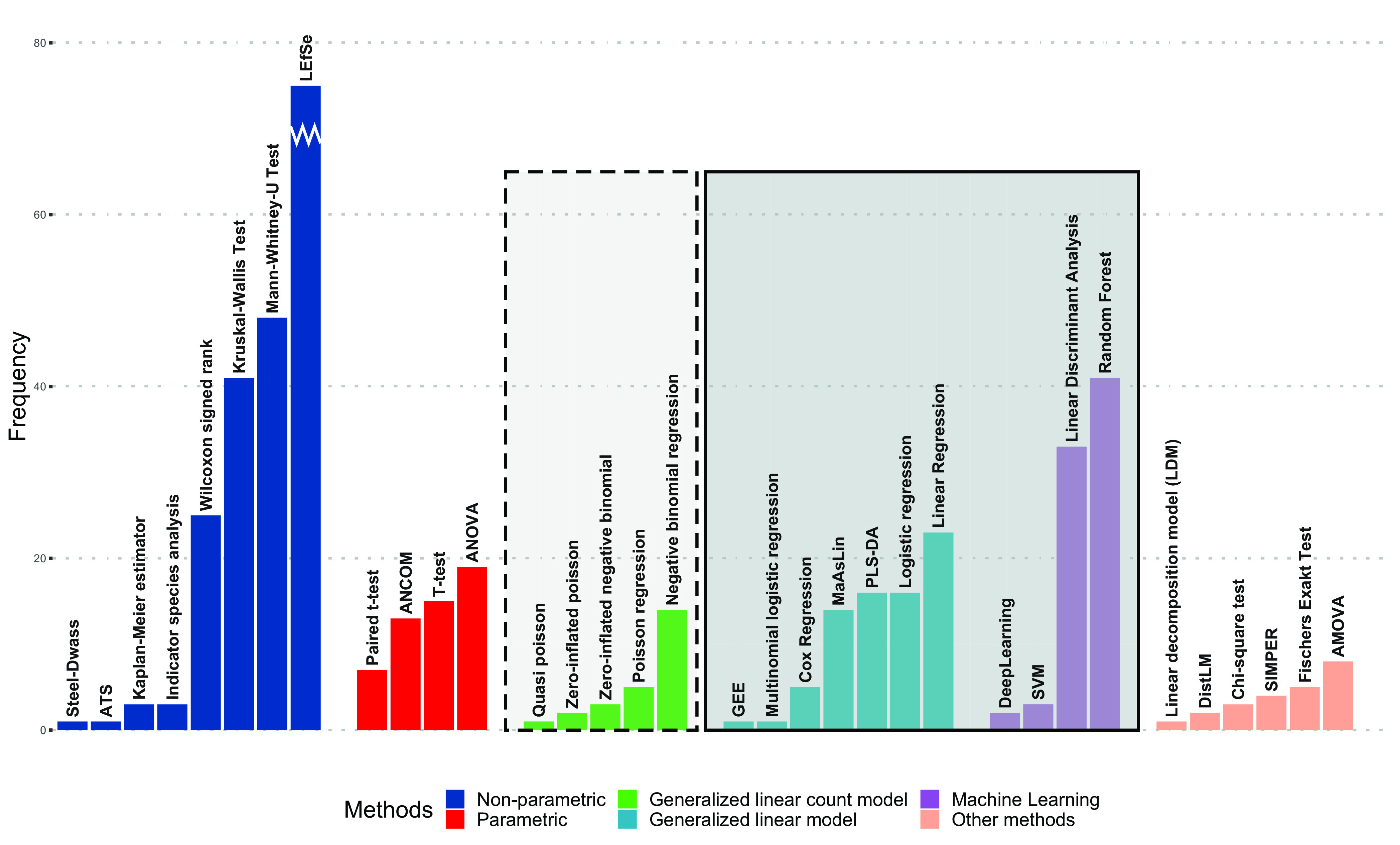
Bar chart of methods used for differential abundance testing grouped by category. Methods highlighted in dark gray model the microbiome as the independent variable. Methods highlighted in light gray model the microbiome as dependent variable. Note that to improve interpretability of the plot, the bar for LEfSe (*n* = 132) was truncated to fit into the scale.

All methods—including those not specifically discussed in the text (28, 51–56)—observed among all studies in this review can be found in Fig. 6.

### Differential abundance analysis with clustered observations.

Among all studies that investigated differential abundance, 113 studies analyzed clustered data. However, most (*n *= 68) of these studies used analysis techniques which are not designed for clustered data. A closer look at these studies revealed that strategies to avoid the direct analysis of nested or longitudinal data were common. Taking LEfSe (36) as an example, 10 out of 22 studies which used LEfSe for the analysis of clustered data treated repeated observations falsely as independent groups, while the rest (*n *= 12) circumvented the longitudinal analysis by splitting the analysis into multiple comparisons at different time points or between different subgroups. Forty-five studies (39.8%) applied methods that take into account additional dependencies in the data, e.g., Wilcoxon signed-rank tests (40%, *n* = 18) or linear mixed effect models (22.2%, *n* = 10). However, Wilcoxon signed-rank tests are specifically designed to analyze data obtained from paired samples (e.g., pre- and posttreatment) and are not suited for any other source of clustering, e.g., longitudinal or multicenter sampling.

### Predictive models.

Predictive models were developed by 18.9% (n = 79) of the studies. Among those studies, 78.5% (*n* = 62) used microbiome data as the independent variables and aimed to predict an outcome, e.g., disease status. If the microbiome was treated as the independent variable, the most frequently used prediction model was a random forest classifier (57) (44.9%, *n* = 31) followed by different types of GLMs (30.6%, *n* = 19). Two studies trained a neural network based on previously detected differential taxonomic units. Among those predictive models with the microbiome patterns as the independent variables, 37.7% used LEfSe for variable selection into the respective models. Few studies (12.7%) aimed to predict changes in specific taxonomic units of the microbiome (as the dependent variable), as the outcome of a specific treatment or condition based on subject matter knowledge. All of these studies used GLMs; however, only one study used a count-outcome-based linear model (zero-inflated Poisson regression), while two studies used MaAsLin (42) to build predictive models, and four studies used linear regression models. Throughout all studies, the predictive performance of models was evaluated by receiver operating characteristic (ROC) curves and the respective area under the curve (AUC). A total of 34% of the studies used internal validation measures (e.g., leave-one-out or k-fold cross-validation); one study validated their findings externally by testing the model on an independent cohort.

## DISCUSSION

The aim of this review was to provide information about analysis strategies currently used in studies investigating the human microbiome. The broad range of methods found among the studies in this review might reflect the lack of consensus on the best approach for analyzing microbiome data. Moreover, our results confirm that the interest in the field moved away from general descriptions of the microbiome to more focused research questions and more sophisticated study designs.

Instead of establishing general associations of diseases to microbial dysbiosis, which may be represented by alpha and beta diversity measures, researchers are interested in identifying single taxonomic units or functional pathways that may serve as a therapeutic target or biomarker for the early diagnosis of diseases. An increasing number of longitudinal studies show that researchers are interested in long-term effects of diseases on the microbiome; moreover, the question whether diseases are a cause or an effect of dysbiosis in the microbiome is of increasing interest. One other field of current research is the use of multiple biosamples obtained from the same individual to detect shared responses among microbiomes of different niches of the human body. These types of studies will result in data with additional layer interdependence due to clustering of samples. Independently of the nature of clustering, observations within one cluster express additional dependencies, which—if not taken into account—may bias the results of statistical analyses. While many of the 155 (37%) studies that analyzed clustered data had specifically designed analysis strategies—which are often characterized by a combination of highly individualized approaches and sophisticated visualization of results—these strategies mainly focused on diversity indices, e.g., alpha diversity dynamics over time or shared alpha diversity responses of multiple body sites within the same subject. In contrast, our results show that testing for differential abundance in more complex research designs is challenging. While some studies did not move beyond diversity analyses, other studies applied alternative analysis strategies avoiding the complex data structure while limiting the possibility of detecting true associations. In some cases, methods were applied that treated clustered observations as independent ones, likely leading to biased estimates and spurious associations.

### Alpha diversity.

Although we observed various alpha diversity indices used in the studies included in this review, the focus on four indices suggests high consensus about how to quantify alpha diversity. Different indices address different domains of diversity so that a combined evaluation of different domains seems useful. However, most combinations observed included multiple indices measuring the same domain. In addition, the vast majority of studies did not describe their choice of indices nor did they interpret the implications. Although 43.4% of the studies used clustered data, the majority of the studies did not apply methods that account for additional dependencies in the data (e.g., random effect models) when assessing alpha diversity. Given that most studies investigated multiple alpha diversity measures, the application of inappropriate methods may lead to an even higher number of false conclusions, as multiple testing leverages these biases. Many commonly used alpha diversity measures, e.g., the Shannon index (16), are nonlinear; statistical inference on mean differences may be biased due to the implied assumption of linearity. Effective species numbers (as estimated by Hill numbers [58]) circumvent this problem as they are defined on a linear scale. Hill numbers serve as a generalization to alpha diversity measures defined by the order q. The Hill numbers of the first three orders correspond to the most frequently used alpha diversity indices—observed species richness (19), Shannon index (16), and (inverse) Simpson index (18). A reasonable strategy for future analyses is to use all three measures, as they cover the range from observed species richness to evenness with different weightings so that their combination provides more information about the true alpha diversity than each single index alone. For this strategy, it is necessary to report all results to avoid publication bias.

### Beta diversity.

Beta diversity measures showed homogeneous patterns with weighted and unweighted UniFrac (20); additionally, Bray-Curtis dissimilarity (21) was used in most studies. Again, these measurements represent different types of beta diversity quantification. Bray-Curtis dissimilarity is a nonphylogenetic dissimilarity measure; it quantifies the dissimilarity between two sample pairs ignoring phylogenetic relatedness. Unweighted UniFrac distances incorporate phylogenetic information as they quantify the fraction of shared branch length on the phylogenetic tree. Its weighted counterpart weights the branch length according to the abundance of the respective taxa. The use of multiple measures that complement each other is found to be common, but only a few studies describe their choices and interpret the respective results in the context of their motivation. However, analogous to the use of complementary alpha diversity measures, it is a reasonable strategy to use all three beta diversity measures and actively interpret detected differences, as these differences may provide useful insight beyond the information that a single metric provides.

Differences in beta diversity measures between study groups were mostly assessed by PERMANOVA (27) or ANOSIM (28). Both methods construct an empirical null-distribution based on permutations of group labels. The permutation scheme needs to be adjusted when analyzing clustered data in order to take the additional dependencies into account, restricting the permutation of group labels within the clusters. In the special case of repeated measurements, additional restrictions may be used to account for temporal dependencies between observations.

### Differential abundance.

The highest level of heterogeneity was observed for differential abundance analysis. The methods used in the studies included in the review suggest that researchers are aware about the violation of assumptions of parametric statistical methods in microbiome studies, as the use of nonparametric models was common.

Only a minority of studies aimed to account for the nature of the data by applying generalized linear models for count data, e.g., Poisson (46) or negative-binomial (45) regression. Although opting for models assuming count-based outcomes may be a reasonable strategy to represent the true structure of the data, these methods are often not specifically designed for high-dimensional data and may produce results that should be interpreted carefully. Although count-based models are able to account for compositionality and various sequencing depth by incorporating the sequencing depth as offset, we could not identify whether studies included these offsets. Hawinkel et al. (59) showed recently that the negative binomial distribution often poorly fits microbiome data. Given the large number of univariate tests in microbiome studies, even a small number of bad fits may influence global inference substantially when corrections for family-wise error rates are applied. A substantial amount of studies still chose to tackle the analysis using linear models assuming normally distributed errors, which are likely to be inappropriate for the analysis of microbiome data without proper preparation of the data, e.g., by applying transformations to account for compositionality. All studies transformed their data to relative abundances where necessary. We could only identify 12 studies that applied transformations beyond the calculation of relative abundances, e.g., log-ratio transformations as referred to by reference 44. Although simple transformation may not be sufficient to deal with compositionality in the data, we could identify only ANCOM (40) as a method used that inherently takes compositionality into account. Although Dirichlet multinomial models are suitable for compositional data as well, no study applied these models to test for differential abundance.

Linear discriminant analysis effect size (LEfSe) (36) was the most frequently used method overall. LEfSe couples a series of standard nonparametric methods—Kruskal-Wallis test (39), Wilcoxon rank-sum test (37, 38), and linear discriminant analysis (56)—to detect differentially abundant taxonomic units and subsequently estimate the effect size for each detected unit. These steps are performed on relative abundances to account for compositionality in the data. As one of very few methods specifically promoted to analyze microbiome data, LEfSe is an established tool that is easily accessible via the Galaxy platform and straightforward to use; it provides appealing visualizations and easily interpretable results. However, as LEfSe applies a series of classical nonparametric methods, its main advantage is to protect false-positive rates, while accepting higher rates of false negatives. Although it may be desirable to reduce false-positive rates, it limits the ability to detect important true effects. When analyzed with classical nonparametric methods, e.g., Kruskal-Wallis tests, the analysis has to be performed univariately taxon by taxon. Instead of applying the method to every single taxon, analysis is often aided by subject matter knowledge, focusing on a small set of taxa of interest. Although this may represent a valid strategy, it is impractical when no subject matter knowledge is available, e.g., when the aim is to detect novel biomarkers, and analyses cannot be focused on single taxonomic units.

The simulation study by Thorsen et al. (60) showed that methods with the lowest false-positive rate also had the lowest predictive performance (and vice versa). Rank-based methods may be limited in their statistical power due to constraints in sparse data. In contrast, parametric models may show higher power but also inflated false-positive rates due to violated distributional assumptions. Although permutation tests may be susceptible to small sample sizes common in microbiome studies and highly zero-inflated data, Thorsen et al. (60) recommend the use of permutation tests or metagenomeSeq (61), which is based on a zero-inflated Gaussian mixture model. Another simulation study by Weiss et al. (8) showed, however, that metagenomeSeq had the highest false-positive rate among all inspected methods. While metagenomeSeq was not used in any of the studies included in this review, permutation tests were mostly applied to distance measures; only a few studies applied permutational methods for differential abundance analysis. Given the frequent use of classic rank-based methods, we would like to argue that permutation tests might be a better alternative. While the application is straightforward in most settings, the results are much more robust to bias and maintain appropriate statistical power. We recommend using permutation tests as a replacement for rank-based methods like Wilcoxon rank-sum tests or Kruskal-Wallis tests, as permutation tests are more flexible, easy to implement, provide higher power, and are easily adjustable for clustered data structures.

### Clustered data structure.

Although 37% of all studies in the review analyzed clustered data, none of them used an adequate strategy to tackle differential abundance analyses in the context of clustered data. Despite the fact that we observed thoroughly designed analysis strategies for longitudinal microbiome data, these studies mainly focused on general microbiome dynamics which can be captured by diversity measures. However, as the field advances, the focus will shift to the complex interplay of individual taxonomic units, either longitudinal or across different body sites. Our results emphasize that currently no methods are used that adequately addresses research questions that move beyond the investigation of microbiome diversity dynamics.

### Conclusions.

The high heterogeneity in methods used for differential abundance analysis implicate a need for a standardized guidance for the analysis of microbiome studies in human hosts in order to improve reproducibility. Guidance documents will improve comparability and reproducibility among studies by requiring researchers to critically think about their design choices and to motivate proactive decision-making. They further motivate researchers to share the intentions and aims of their analyses, improving the interpretability of the presented results. So far, no such guidance for human microbiome studies is available; however, a recent publication by Calle et al. (62) may serve as a template for the analysis of microbiome data. Considering the results in this review, we summarized a collection of reasonable strategies for future research in Table 3 that prevent many of the possible pitfalls.

**TABLE 3:** Recommendations^*a*^ for future research based on the most commonly identified pitfalls in this review

Subject	Recommendation^*b*^	Rationale
Alpha diversity	Hill numbers (58) of first three orders	Linear scale, quantify different information, represent most commonly used diversity indices, combination provide more information compared with single indices
Beta diversity	Bray-Curtis dissimilarity (21), unweighted UniFrac, weighted UniFrac (20)	Most commonly used diversity indices, quantify different information, combination provide more information than single indices
Differential abundance	Replacement of common nonparametric methods (e.g., Wilcoxon rank-sum) by permutation tests	Robust, higher power, easy to implement, easily adjustable to account for clustered observations, results more directly interpretable than rank-based methods
	Model-wise assessment of fit/violation of assumptions	Generally well fitting models may show bad fit for some taxa; misfit of univariate models may influence other analyses, e.g., due to FDR^*c*^ corrections
	Triangulation	The use of multiple methods, which are ideally susceptible to different data characteristics, may protect from false-positives; reasonable if very conservative approach is needed, e.g., if appropriateness of methods is unclear/not known
General	Careful consideration of data structure, especially due to study design	Ignoring the underlying data structure jeopardizes the meaningful interpretation of results; detecting dependency structures is a matter of subject-matter knowledge, e.g., due to the study design

a Note, that these recommendations represent sensible strategies based on current knowledge to easily avoid common pitfalls.

b References are in parentheses.

c FDR, false-discovery rate.

We summarized the main shortcomings with respect to microbiome data in Table 4. This table emphasizes the ability of models with microbiome as outcome to account for microbiome-specific data characteristics and indicates the main challenges in microbiome data and possible bottlenecks with respect to the most frequently used types of methods.

**TABLE 4:** Ability of models with microbiome as outcome to account for microbiome-specific data characteristics

Statistical approach	Overdispersed	Zero-inflation	Compo- sitionality	Multivariate outcomes	Adjusting for confounders	Extension to clustered data	Extension to longitudinal data
Nonparametric models^*a*^	No	Indirect^*b*^	Indirect	Yes	Not possible	Easy	Easy
Parametric models^*c*^	No	Indirect	Indirect	Yes	Possible	Easy	Easy
Linear regression	No	Indirect	Indirect	Indirect	Possible	Easy	Easy
Poisson regression (46)	No	Indirect	Indirect	No	Possible	Easy	Easy
Negative binomial regression (45)	Yes	Indirect	Indirect	Indirect	Possible	Easy	Easy
Zero-inflated Poisson regression (47, 48)	No	Yes	Indirect	No	Possible	Difficult	Difficult
Zero-inflated negative binomial regression (48)	Yes	Yes	Indirect	Indirect	Possible	Difficult	Difficult
ANCOM (40)	Yes	Indirect	Yes	No	Possible	Easy	Easy
Dirichlet-multinomial regression (67)	Yes	Indirect	Yes	Yes	Possible	Difficult	Difficult
LEfSe (36)	No	Indirect	Indirect	No	Not Possible	Easy	Difficult
MaAsLin (42)	No	Indirect	Indirect	Indirect	Possible	Easy	Easy

a Nonparametric refers to group comparison models like Kruskal-Wallis and Wilcoxon signed-rank test.

b Indirect indicates that additional adjustments or preprocessing steps are necessary to take a specific characteristics into account.

c Parametric refers to group comparison models like *t* test and ANOVA.

In order to construct more sophisticated guidelines, independent simulation studies are crucial for benchmarking methods on a large scale with respect to the full complexity of microbiome data. So far, only a few independent benchmarks are available. Despite the discussed simulation by Thorsen et al. (60), to the best of our knowledge, only two recent simulation studies evaluated a collection of frequently used methods. The results by Hawinkel et al. (63) indicated excess false discoveries among all investigated methods, independent of the chosen benchmarking tool. While Weiss et al. (8) mainly focused on the effects of normalization techniques on differential abundance testing, the results showed that benchmarking results are highly dependent on the chosen simulation strategy. Although a wide range of other simulation studies are available, these simulations were intended to justify newly developed methods and inherently favor the proposed method, e.g., by simulating data from the same parametric model as the method is based on. As currently no further independent evaluation of the performance of methods is available, the magnitude of bias induced by the use of inappropriate methods is not known. The results may aid researchers in making informed choices regarding appropriate methods and analysis strategies needed to adequately address their research questions.

Furthermore, there is a clear need for novel methods designed to analyze microbiome data obtained by more complex study designs. Independently of the statistical properties of new methods, it is crucial to provide these methods in an accessible and transparent way. The most frequently observed methods share the characteristic that they are straightforward to apply, are well documented, and provide accessible and interpretable output. Although oversimplification of complex methods should be avoided, transparent documentation, including extensive tutorials is crucial.

## MATERIALS AND METHODS

**FIG 7:**
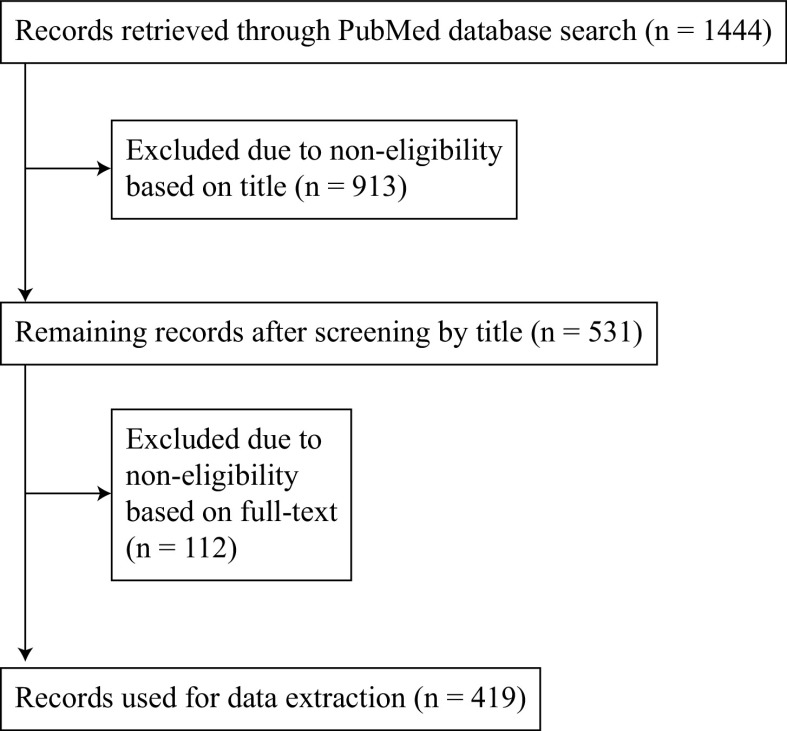
Flowchart of literature review and data extraction process.

We identified relevant studies published in peer-reviewed journals via search of the PubMed database using the search term “(16s[All Fields] AND rrna[All Fields]) OR amplicon[All Fields] OR shotgun [All Fields]” with the filter “Humans.” Publications from June 2018 to June 2019 (the date of this PubMed search) were screened. Primary studies that used 16S rRNA or metagenomic shotgun sequencing and that investigated human subjects were included. Methodological studies, reviews, pooled analysis of published studies, studies in mice, and studies that investigated viruses or eukaryotic organisms were excluded. Each of the three reviewers (S.K.B., N.R., and T.B.) extracted data from the included studies regarding the following domains: study design, research question, sample characteristics, data characteristics, software, and statistical methods. The workflow is depicted in Fig. 7, while an overview of all included and excluded publications can be found in Table S1 in the supplemental material. The extracted data were compared between the reviewers and screened for anomalies (e.g., if categories were interpreted differently so that one reviewer assigned that category more often than the other reviewer). In case differences were detected, they were discussed and data extraction was revised and repeated for the respective categories. In total, information from 419 publications was extracted.

10.1128/mSystems.01154-20.3 TABLE S1 All publications that fulfilled the inclusion criteria for the review, as well as those publications that were excluded and the respective reason of exclusion. Download Table S1, XLSX file, 0.2 MB. Copyright © 2021 Kleine Bardenhorst et al. 2021 Kleine Bardenhorst et al. https://creativecommons.org/licenses/by/4.0/ This content is distributed under the terms of the Creative Commons Attribution 4.0 International license.

In the domain “research question,” we extracted information about the objective of the study (as described in the publication) and the actual analysis performed to investigate whether the chosen analysis strategy was adequate to answer the respective research question. Objective and actual analysis were each categorized as either (i) descriptive if no inferential statistical analysis were (to be) performed, (ii) analytical if at least in one part inferential statistical analysis was (to be) used to answer the research question, (iii) predictive if any prediction models were (to be) built (including an assessment of their predictive performance), or (iv) assessment of treatment effects if such an effect was clearly defined as the outcome of interest. We assigned each study to one main objective. If a study met several objectives, the most advanced one (in the order treatment effect, predictive, analytical, and descriptive) was selected.

We further inspected which taxonomic levels were used for microbiome analysis. The levels of taxonomy are phylum, class, order, family, genus, and species, with phylum and species representing the highest and lowest level, respectively. In studies using 16S rRNA or metagenomic shotgun sequencing, these taxonomic levels are generally obtained in the following way: the reads from the sequencing step are clustered by applying a predefined similarity threshold (often 0.97) into operational taxonomic units (OTUs) to control for random variations due to sequencing errors. These OTUs are aligned to a reference database to infer taxonomic annotations of each respective OTU. Dependent on the underlying sequence, OTUs can be assigned to a specific taxon at a taxonomic level (often genus). Higher taxonomic levels can be constructed as the sum of lower taxonomic levels, with species being grouped into genera, which are then grouped into families, and so on. However, OTUs have been criticized in recent literature, mainly due to their lack of biological interpretability. Callahan et al. (64) proposed an alternative approach, referred to as (exact) amplicon sequence variants (ASVs). Regardless of the use of OTUs or ASVs, analyses can be performed at every level of this taxonomy. As the higher taxonomic ranks are accumulated from lower ranks, dimensionality and proportion of zero counts decreases with every step upward the taxonomy. However, given that the lowest level provides the most detailed information (e.g., about pathogenic species), avoiding lower levels may limit the possibility to detect important associations.

As there is plenty of software available for the analysis of microbiome data, we explored which software packages were used most frequently. We focused on the software used for statistical analysis and not the bioinformatics pipeline used for any preprocessing of the data. If, however, a bioinformatics pipeline was used for statistical analysis independently of data preprocessing, it was included in the domain software.

The first step in the analysis workflow of microbiome studies is usually the assessment of alpha and beta diversity. Alpha diversity is defined as the diversity within a given sample, generally measured by the dimension richness (number of observed taxa) and evenness (equality of distribution across observed taxa). Note, that we grouped the Simpson index and the inverse Simpson index together, as they can be directly convertible into each other. Beta diversity is defined as the diversity between samples. As various measures exist for both alpha or beta diversity, we assessed which measures were used and whether only one or multiple measures were used. Given that most measures differ in their definition of diversity (both for alpha and beta diversity), we also investigated which set of measures were used together most frequently.

Ordination methods are typically applied to beta diversity measures to visualize underlying patterns in the data. Principal-component analysis (PCA), often used in other fields, is based on Euclidean distances and has therefore been deemed inappropriate for microbiome data; instead, alternatives like the principle coordinate analysis (PCoA) (30) or nonmetric multidimensional scaling (NMDS; rank-based) (30) are used. Although applied to beta diversity measure as well, ordination in NMDS is based on ranks instead of the raw distances and may produce visually more interpretable results than PCoA.

Clustering techniques are another way to structure microbiome data without a predefined hypothesis (unsupervised), e.g., to derive biological clusters (like so called “enterotypes” [65] in the gut). While some unsupervised clustering techniques are adapted for the use in microbiome data and are used in combination with a preceding transformation of the data (like the k-means algorithm), others (e.g., Dirichlet multinomial mixtures [31]) are specifically designed for microbiome data and applied to raw count data directly.

If there is an a priori hypothesis, supervised approaches to detect differences in beta diversity between predefined groups can be applied. Methods include permutational multivariate analysis of variance (PERMANOVA) (27) and analysis of similarity (ANOSIM) (28). PERMANOVA and ANOSIM are both distance (or dissimilarity)-based permutation methods (using beta diversity measures) designed to mimic an analysis of variance (ANOVA), without assuming a normal distribution of errors. While PERMANOVA is applied directly to the distances, ANOSIM first assigns ranks to interindividual distances. Analogous to the assumption of homogeneity of variances in classical ANOVA, both methods assume heterogeneity of multivariate dispersion. While PERMANOVA is quite robust against violations of this assumption given balanced study groups, violations will inflate type-1 error rates for ANOSIM. If a study compared two groups with PERMANOVA or ANOSIM, we extracted the sizes of these groups and calculated their ratio as a measure of balance. Although PERMANOVA is not sensitive against unbalanced study designs, heterogeneity in dispersion among groups will result in biased estimates for both methods (66) if the study groups are not balanced.

Finally, we extracted information on which methods were used to test for differential abundance of single taxonomic units. As this stage of the analysis poses the most complex challenges, there is currently no consensus about appropriate methods. An overview of the most frequently used methods for microbiome analysis and whether they deal with the characteristics in microbiome data is displayed in Table 4 in the discussion section. Only if indicated with “yes” is the respective characteristic addressed by the method directly. Several characteristics can be accounted for by preprocessing the data or extending methods (indirect). However, this does not ensure that the approach is adequately used in practice. An extensive simulation study by Thorsen et al. (60) evaluated the performance of frequently used approaches; based on their results, they advise the use of permutation tests over rank-based or parametric approaches that assume count distributions (e.g., negative binomial). According to the authors, permutation tests outperformed other methods because they are less limited in statistical power (compared with rank-based approaches) and do not make any distributional assumptions which are likely to be violated due to the characteristics in microbiome data. However, permutation methods do not address all characteristics directly, e.g., compositionality or zero-inflation. We extracted detailed information about how differential abundance analysis was performed and whether the findings of this simulation study are applied in current practice. Methods were categorized as either nonparametric, parametric, generalized linear models for normally distributed or binary outcome data (GLM), generalized linear models specifically for count data (GLMc), and other methods that did not clearly fit into one category; they were mostly based on distance measures in combination with permutation tests. An overview of these classifications can be found in Fig. 6. If the objective of the respective study was prediction, we assessed which type of models were used (now including machine learning methods like random forests and support vector machines) and how their performance was measured.

Among all stages of the analysis—alpha diversity, beta diversity, and differential abundance testing—we specifically looked at how analysis strategies were adopted in case of clustered data. If clustering is present in the data, observations within a cluster are expected to be more similar compared with observations between clusters. Ignoring these additional dependencies will induce bias and possibly lead to spurious associations. Therefore, we grouped methods according to whether they took into account these dependencies adequately.
